# Note on Blood-Letting without Loss of Blood

**Published:** 1895-04-27

**Authors:** A. Hanbury Frere

**Affiliations:** Medical Officer, West Riding Asylum Extension, Menstone


### Medical Progress and Hospital Clinics.

[*The Editor will be glad to receive offers of co-operation and contributions from members of the profession. All letters should be addressed to* The Editor, The Lodge, Porchester Square, London, W.]

### NOTE ON BLOOD-LETTING WITHOUT LOSS OF BLOOD.

By A. Hanbury Frere, M.B., Medical Officer, West Riding Asylum Extension, Menstone.

The practice of "blooding," at one time so universal and so wide in its application, has to most of us become a thing of the past. At the beginning of the century when antiphlogistic treatment was supposed to "jugulate" the disease, the physician did not hesitate to draw from the arm many ounces of blood, and the death of the patient was attributed to the want of early and bold venesection.

Now all this is changed. But although we do not actually bleed our patients, we very frequently indeed make use of this method in principle, if not in practice.

The theme which I bring forward is not a new one, but has been suggested to me by the study of a paper by Professor Chiene.[1] The principle involved is a most important one, and will be found of the greatest value. We leave college equipped with all that the text-book can supply, ready to diagnose and to treat any typical case. When we come into practice we find that our cases are, for the most part, atypical, and often exhibit combinations of diseases, as Roberts has so well pointed out in the Lettsomian lectures.[2] Hence the necessity of a wide and comprehensive view in medicine, hence also the value of principles.

Disease has been called the misplacement of vital forces, and is to all intents and purposes an injury. When a part is injured there is an increased flow of blood to it, resulting in an increased deposit, and finally an increased production at the injured part. These phenomena which take place in the organ or tissue that is injured are largely due to an alteration in the vascular supply of the part, and are in every way identical with the changes observed in inflammation. It is necessary that I should briefly mention these modifications in diseased or injured tissue, because they have an important bearing on the subject of this paper. For if the vascular system plays such an important part in the changes observed in disease, I hold that it is upon the vascular system that we must bring our influence to bear in our efforts to bring about recovery.

We may with advantage, I think, look upon a diseased organ or tissue as an injured part, and I propose to consider very briefly a few instances in which our treatment of disease depends very largely upon a relief of vascular tension. As Chiene points out, the amount of nerve force and blood supply in the body at any given time are fixed quantities. If then an extra amount of nerve force or blood supply be expended at any part it must be drawn from some other part. The application of an irritant will, through the nervous and vasomotor systems, have an effect upon other and often distant parts, causing a change in the vascular supply. This is, briefly, the action of counter-irritation. Either directly, or through the intervention of the nervous system, a counter-irritant acts by relieving vascular tension in the affected part which we may wish to influence. It may, therefore, be looked upon as a form of blood-letting. In the same way not only purgatives, but also diuretics and diaphoretics will exercise their beneficial influence by a relief of vascular tension or blood-letting.

The employment of counter-irritation has largely displaced leeching, scarifying, and general blood-letting, and its application is far wider than the old form of bleeding. Indeed, unlike venesection, its indication is by no means confined to acute arterial conditions or inflammations. Once we recognise the principal of relief of vascular tension it is easy to multiply examples of how we may in this way counteract the various derangements with which we may have to deal.

The instances we have so far considered bring about a relief of vascular tension by withdrawing blood from the affected part, either directly or indirectly, but always by a *vis a fronte*. Such also is the action of nitrites, which, by dilatation of the capillaries at the periphery, or by overcoming various spasmodic affections of the arterioles, will relieve central engorgement. But when an organ is acutely inflamed we may desire to restrain the rush of blood to the part by such remedies as digitalis, aconite, and ipecacuan. Lastly, we may require to relieve vascular tension, more especially when there is stasis in the veins by means of a *vis a tergo*, such as stimulants, tonics, and belladonna.

When we come to consider disease from the point of view that I have indicated, paying special attention to the state of the circulation, we shall find that our cases fall naturally into certain fairly well-defined groups, depending upon the part of the vascular system that is chiefly involved in the disturbances occurring in diseased or injured tissues. These groups will, I believe, be found to be mainly two, depending on whether the hyperæmia be *active* or arterial, as in acute inflammatory affections; or *passive* or venous.

As I hope at some future time to enter into this subject more in detail, I will not say more than that as the symptoms and signs of these two classes are well-defined, so the treatment is exactly the opposite in each. I am aware that, in the opinion of most, disease depends "primarily upon modifications in the processes of cells, rather than of the fluid whence cells obtain their nourishment,"[3] and that more good is to be got by a study of the tissues than from a study of the blood. Yet, as our only means of influencing the tissues by our treatment is through the blood stream, I hold that by paying attention to the state of the circulation in the way that I have indicated, we shall not only arrive at a much clearer diagnosis, but our treatment will be based upon much more rational principles than those involved in the so-called "expectant treatment." I cannot do better than conclude by quoting Sir B. W. Richardson, who says[4] that in relieving the lungs from congestion "we must look primarily to the action of the heart and the volume of the blood with which it is charged." In other words, "we must either, by increasing the tonicity of the heart, cause the blood to flow over the pulmonary circuit with greater rapidity and completeness, or we must reduce the volume of the column of blood so that the lesser circulation may be subjected to practical venesection through the general circulation."

[1] American Practitioner, 1879. [2] B. M. J., Jan. 26, 1895. [3] Gamgee Physiological Chemistry, vol. I., 1880. [4] Hospital, Jan. 26, 1895.

